# Gatekeepers in primary care - a qualitative study on manual therapists’ role in triaging patients with back and neck pain

**DOI:** 10.1186/s12913-025-13593-w

**Published:** 2025-10-27

**Authors:** Iben Axén, Elias Schriwer, Jesper Hjertstrand, Eva Skillgate, Per J. Palmgren, Andreas Eklund

**Affiliations:** 1 Unit of Intervention and Implementation Research for Worker Health, Institute of Environmental Medicine (IMM), Karolinska Institutet, Nobels väg 13, Solna, Stockholm, 171 77 Sweden; 2 Department of Learning, Informatics, Management and Ethics, Karolinska Institutet, Widerströmska huset, Tomtebodavägen 18A, Solna, Stockholm, 171 65 Sweden; 3 ELIB, Et liv i bevegelse, The Norwegian Chiropractic Research Foundation, Oslo, Norway; 4 Musculoskeletal & Sports Injury Epidemiology Center, Department of Health Promotion Science, Sophiahemmet University, Box 5605, Stockholm, 114 86 Sweden

**Keywords:** Chiropractic, Manual therapy, Low back pain, Neck pain, Qualitative research, Focus groups, Community health centers, Scope of practice, Interprofessional relations, Evidence-based practice, Reconstructing

## Abstract

**Background:**

In Sweden, almost 60% of patients seeking care at primary healthcare centers do so due to musculoskeletal disorders. These patients constitute a strain on the healthcare system. Manual therapists are experts in diagnosing patients with these conditions but are usually not part of healthcare centers in Sweden. Further, there is a significant societal problem with overuse of medications, injections, medical imaging, and unnecessary surgeries, so-called low-value care, for back and neck pain. We aimed to explore the current management of patients with back and neck pain in primary healthcare centers, staff’s perceptions regarding the need for improvement, and incentives and concerns about having licensed chiropractors and naprapaths (manual therapists) triage these patients as part of the primary healthcare center team.

**Methods:**

In this qualitative study, we conducted semi-structured focus group interviews with 20 participants employed at three primary healthcare centers in the Stockholm region, chosen based on location, organization, and exposure to manual therapists. Participants were purposefully selected from different professions. Data were analyzed using inductive qualitative manifest and latent content analysis.

**Results:**

The current management of patients with back and neck pain was described in terms of long waiting times and patients’ expectations of seeing a medical doctor and receiving imaging. Thus, there was room for improvement by reducing low-value care. Participants had limited knowledge about the competence of chiropractors and naprapaths and were concerned about the boundaries of professional identity. Decreased physician workload and more choices for patients were mentioned as incentives for manual therapists to take on a triaging role.

**Conclusions:**

Staff at three primary healthcare centers in the Stockholm area described the current management of patients with back and neck pain as navigating the expectations of patients and their overreliance on seeing their physician, not other caregivers, resulting in long waiting times. Alongside patients expecting imaging, these were incentives for chiropractors and naprapaths triaging these patients as an opportunity to improve care. A lack of knowledge about the competence of manual therapists was a concern, and assigning manual therapists to the primary healthcare team would necessitate a reconfiguration of the care process, potentially challenging the boundaries of professional identity.

**Supplementary Information:**

The online version contains supplementary material available at 10.1186/s12913-025-13593-w.

## Background

Primary healthcare worldwide is facing a major challenge in addressing patients with back and neck pain [1, 2]. These are prevalent disorders [2] that most commonly should not be prescribed imaging, strong medications (such as opioids), nor surgery, described as low-value care. Most of these patients should be managed with reassurance, exercise, and some with manual therapy [3].

In Sweden, publicly funded primary care is challenged to meet patient demand on many fronts. The National Board of Health and Welfare and the National Health Competence Council have, in a report from 2022, raised concerns about the lack of resources in the primary healthcare sector where 19 out of 21 regions in the country have indicated that the supply of physicians, nurses, psychologists, physiotherapists, dietitians, and occupational therapists fail to meet the demand [4]. In Sweden, all citizens’ first line of healthcare is through a publicly funded primary healthcare center (PHC), where physicians work alongside nurses, and sometimes physiotherapists and dieticians.

According to a recent survey, nearly 60% of patients seeking care at PHCs in Sweden do so due to musculoskeletal disorders [5], most commonly back and neck pain. In studies of the management of musculoskeletal disorders and low back pain, patients seen by physicians often receive recommendations inconsistent with clinical practice guideline recommendations [6, 7], e.g. pharmacological treatment or referral for surgery. Survey and registry data have also shown that care received by individuals with osteoarthritis in primary care settings is variable and often inconsistent with clinical practice guideline recommendations [8–10].

Manual therapists in Sweden, in this project licensed chiropractors and naprapaths, have been part of the Swedish Health and Welfare system for more than 30 years. Despite this, these caregivers are unused resources as they seldom work in PHCs. Most manual therapists work privately outside the government-funded healthcare system and typically care for resourceful patients who can afford to pay for their care. Their expertise lies in differential diagnosis and management of musculoskeletal conditions, focusing on evidence-based non-pharmacological interventions.

To address the future health challenges of managing patients with back and neck pain, we must utilize all available resources. Licensed chiropractors and naprapaths have diagnostic competence and are well-suited to triage and manage patients with musculoskeletal conditions, including back and neck pain, as first-contact healthcare providers. To fully leverage this competence, these manual therapists could be designated as first-contact providers within PHCs, thereby reducing the burden of back and neck pain in the population by enhancing adherence to evidence-based guidelines, alleviating the workload on physicians, and minimizing low-value care. Most patients need reassurance, explanations, and advice to stay active, and some should be directed to appropriate care, a physiotherapist, manual therapist, or psychologist. Few patients need to see the physician for further investigation (imaging, blood tests, or specialist referral) or sick leave.

### Aim and research questions

The overall aim of this study was to explore how patients with back and neck pain are currently managed at three PHC in Stockholm, and to examine staff perceptions regarding the need for improvement, and the potential role of licensed chiropractors and naprapaths in triaging such patients within PHC settings. Based on this aim, three research questions were formulated:

1. How are patients with back and neck pain currently managed at the PHCs, and do staff perceive a need for improvement of this management?
2. What are PHC staff perceptions of licensed chiropractors and naprapaths in a potential triaging role for patients with back and neck pain?
3. What concerns and incentives do PHC staff associate with introducing licensed chiropractors and naprapaths in a triaging role for patients with back and neck pain?

## Methods

### Design and positioning of the study

This qualitative interview-based investigation represents the initial phase of a broader research initiative employing a prospective, mixed-methods design grounded in a pragmatic research tradition. This study is best described as an exploratory qualitative study, designed to capture variation across settings and inform the larger project. The current study was situated within an interpretative paradigm, which posits that knowledge is inherently relative and socially constructed [11]. Consistent with this epistemological stance, the research was guided by the assumption that findings are shaped through the dynamic interaction between the phenomenon under scrutiny and the researchers themselves, rather than though discovery of an objective, singular truth [12]. A qualitative research approach was deemed appropriate given the investigators´ focus exploring human experiences [11]. The study adhered to the Consolidated Criteria for Reporting Qualitative Research guidelines to ensure methodological rigor and transparency [13].

The study was informed by the theory of Communities of Practice (CoP), which was used both to frame the phenomenon under investigation and more importantly, as a lens through which to interpret the findings as they were shaped. CoP is a concept developed by Jean Lave and Etienne Wenger [14, 15]. It describes how communities are formed around groups of individuals who share a common interest or concern and seek to deepen their expertise and knowledge in a particular discipline or subject area [16]. The key elements of a CoP include the *domain*, which refers to the shared area of interest or expertise that unites the members, such as a medical specialty, a care model, or patient management. The *community* aspect involves members building relationships through collaboration, discussion, and the exchange of experiences. The *practice* element encompasses the sharing of knowledge, techniques, tools, and best practices aimed at improving their work.

Wenger et al. [16] postulate that the community can serve as vehicle for collaboration, allowing its members to engage in dynamic and meaningful relationships with peers and others. A PHC can be viewed as a CoP, as it brings together professionals who work collaboratively to provide care, develop expertise, and solve problems. From this perspective, it is possible to explore how knowledge is shared and developed within the organization, how different professional roles interact and influence other’s work, and how new members are introduced and become integrated into the shared practice. In the current study, the CoP framework was applied during the analytical phase to interpret how shared understandings, informal learning, and collaborative routines among professionals shaped triage practices. The framework was not used to inform the development of the interview guide; rather, it was introduced later in the process when patterns in the data suggested a meaningful connection to CoP concepts.

### Research team and reflexivity

The multidisciplinary research team comprised six members —two women and four men, none of whom had previous engagement in the study PHCs. Two members (ES and AE) were directly involved in data collection. ES, a medical doctor and PhD candidate, with prior experience in conducting qualitative interviews, served as the primary interviewer. AE, a chiropractor and researcher, attended the interviews in an observational capacity and took field notes. The remaining four team members (IA, JH, EvS and PJP), all manual therapists, and part of a larger research initiative to investigate the integration of manual therapists into PHCs, had experience in qualitative research, with three having substantial experience and one with extensive expertise. The team´s diverse professional backgrounds and research experiences may have influenced the interpretation of the data; however, this diversity also contributed valuable perspectives to the exploration of the phenomenon under investigation.

### Setting

The study was conducted in three large (20–40 physicians and 25000–75000 patient visits annually) PHCs in the Stockholm region in the fall of 2024. These PHCs were purposefully chosen based on location, organization and exposure to licensed chiropractors and naprapaths. Thus, one of the PHCs was located centrally in Stockholm, whereas the other two were in two different suburbs of the city. All were publicly funded. One was a so-called academic PHC, where the staff was used to conduct research in a primary care setting. One PHC collaborated closely with a rehabilitation unit with a manual therapist on staff, whereas the other two did not. Before study start the research team introduced the research project through written information to the senior managers, research managers, and one of the physicians in each PHC.

### Participants and recruitment

We conducted semi-structured focus group interviews, and the choice of this format was based on logistical constraints at the participating primary healthcare centers (PHCs), where staff availability for individual interviews was limited. The PHCs were responsible for recruiting study participants and providing an office for the on-site interviews. A purposeful and maximum-variation sampling strategy was employed to obtain richness and variation in the data from different professional perspectives (e.g., ensuring that each focus group consisted of physicians (at least 50%), nurses, physiotherapists, and management). The sole inclusion criterion was that the participant had to be employed at one of the included PHCs [17].

Twenty staff members from various professions working at the three PHCs participated in the focus group interviews; one interview was conducted per PHC. The participants were diverse in terms of sex (12 females and 8 males), age (27–70 years), and professional experience (1–30 years). Half were medical doctors, and also nurses, managers, and a physiotherapist were included. Their characteristics are presented in Appendix 1.

The research team developed a preliminary interview guide (a translated version is attached as Appendix 2) based on two criteria: the interview should (1) correspond to the aim of the study and (2) use empirical findings from the scientific literature. The CoP framework was not used to inform the interview guide. The focus group discussions were facilitated by ES using the semi-structured interview guide to ensure consistency across groups while allowing for open dialogue. A second researcher, AE, was present to take field notes and support the facilitation process as needed. Each interview was initiated with a presentation of the interviewers, general information about licensed chiropractors and naprapaths and the study’s aim. Semi-structured interviews with open-ended questions enable study participants to raise various and sometimes contradictory aspects of the studied phenomenon and contribute to a deep and nuanced understanding of the research questions. No repeat interviews were conducted. The interviews were audio-recorded using a laptop microphone with recording software, and a mobile phone was used as a backup. All interviews were conducted, transcribed, and analyzed in Swedish. One research team member translated the quotes into English, and they were then checked by the other researchers for accuracy.

### Data analysis

A professional transcription service transcribed the interviews verbatim. The audio-recorded sessions averaged 45 min in length (49, 49, and 38 min) per group.

The data were analyzed using an inductive approach to qualitative content analysis [18–20], primarily guided by the method outlined by Graneheim and Lundman [20]. While the analysis was conducted with an inductive orientation, elements of abductive reasoning were also present, as the theoretical lens of CoP informed the interpretation of findings. This allowed for iteratively moving between empirical data and theoretical concepts to deepen our understanding of the patterns that emerged. Transcripts were examined line by line, and subcategories and categories were developed without the use of predetermined coding schemes. The analysis comprised several steps: (i) the transcribed interviews were initially read by JH and ES to gain familiarity with the content; (ii) notes were made in the text and transferred to an Excel spreadsheet; (iii) the textual data were read and analyzed by all authors, both individually and collaboratively; (iv) JH identified meaning units related to the study´s the aim and the interview guide questions; (v) the meaning units were discussed and condensed, and codes relevant to the phenomenon under investigation were developed by JH, EJ, and AE; and (vi) an interpretative analysis was conducted, extending beyond the explicit manifest content. Categories were interpreted and developed into themes that reflected the underlying latent content of the data [20]. During stages v) and vi), it became evident that the three focus groups, consisting of 20 participants in total, provided sufficient “information power” [21].

After completing the analysis, the main findings — consisting of thematically labeled textual content with supporting quotations — were translated into English. During multiple consensus meetings, the translated material was reviewed by the research team. The condensed English versions were compared with the original Swedish transcripts to ensure that the intended meaning was preserved.

One way to conceptualize these analytical layers is through the communication theory axiom proposed by Watzlawick et al. [22], which distinguishes between manifest and latent content. Manifest content refers to what the text explicitly states —its surface structure and most apparent meanings. Conversely, latent content involves an interpretative reading of what the text implicitly conveys, capturing deeper structural meanings. The entire analytical process was discussed and refined until a consensus was reached among the researchers. Although the above steps might seem sequentially ordered, the analytical process and search for patterns were rather dynamic, iterative, and recursive. An Excel spreadsheet was used to organize and manage the qualitative data, allowing for systematic categorization and efficient identification of themes and key patterns.

The study’s trustworthiness was strengthened through investigator triangulation. From the outset, all team members were actively involved in the planning, data analysis, and writing phases, ensuring a diversity of perspectives throughout. A detailed description of the study design and data collection methods was provided to support dependability. Data stability was maintained by repeatedly reviewing the transcripts and engaging in discussions until consensus was achieved. Credibility was further enhanced by the collective participation of all team members in the data analysis [23], and the inclusion of representative quotations from the transcripts [24]. Throughout the analysis, continuous comparisons were made between the subcategories, categories, and the original data transcripts. This process was particularly informed by the senior investigator’s extensive understanding of the empirical context, ensuring strong alignment between the data and the findings. Patton’s criteria of internal homogeneity and external heterogeneity [17], were carefully followed, ensuring consistency within categories and clear distinctions between them.

### Ethical considerations

Information about the study was emailed to the director at each PHC, and the same information was reiterated verbally prior to each focus group interview. Participation was voluntary, and participants were informed that they could withdraw at any time. Written informed consent was obtained before the interviews, and verbal consent was also recorded to ensure participants fully understood their rights. Full confidentiality was assured, and no identifiable information was collected, thereby ensuring data anonymity. The study was conducted in accordance with the tenets of the World Medical Association Declaration of Helsinki. As no personal data were collected, the project was deemed not to require ethical approval by the Swedish Ethical Review Authority (Reference number 2024-05148-01).

## Results

The analysis generated 21 units, which were further condensed into six categories and three overarching themes (see Table 1). The categories and themes describe the participants’ understanding of how patients with back and neck pain are currently managed at the PHC, as well as staff perceptions regarding the need for improvement and the potential role of licensed chiropractors and naprapaths in triaging patients with back and neck pain at PHCs.

**Table 1:** The units, resulting categories, and themes resulting from 3 focus group interviews with PHC staff about introducing manual therapists to triage patients with back and neck pain

Units	Categories	Themes
Patients want to see a physician rather than other health care professionals	**In search of the silver bullet**	**Navigating the weight of expectations**
Patients are keen to explore extensive diagnostic imaging		
Patients look for a quick fix		
Hesitation among patients to visit a MT rather than the physician		
Long waiting times for patients	**Left waiting alone**	
Responsibility of the care placed on the patient		
Desire among PHC staff to learn more about MTs	**Bridging the gap**	**Reconfiguring the care process**
Increase the knowledge about MTs among patients		
Increase the confidence that PHC staff has for MTs		
Reduce physician workload	**Easing the** ** burden**	
Fewer patient visits overall at the PHC		
Avoid unnecessary prescriptions and expensive diagnostic tests	**Avoidin** **g low-value care**	
Lack of physical space to accommodate the MT	**Space and time as precious commodities**	
Time consuming dealing with the large group of patients with back and neck pain		
Lack of communication between health care professionals in general	**Shaky foundation for future collaboration**	**Challenging boundaries of professional identity**
Large variability in clinical skills and competency between different MTs		
Lack of knowledge among PHC staff regarding the work and knowledge that MTs possess		
Lack of professional skill and competence of the MT		
Large overlap in area of expertise as well as lack of distinction regarding scope of practice between different MTs	**Blurred professional lines**	
Risk of mid-level encroachment where the MT takes over responsibilities that are traditionally associated with physicians		
Risk of missing severe illnesses		

MT = manual therapist, i.e. licensed chiropractors and naprapaths, PHC = Primary healthcare center

### Theme 1: Navigating the weight of expectations

This theme emphasizes how patients’ expectations influence the current delivery of care. PHC staff described how patients frequently seek out physicians as their first and preferred point of contact, expecting medical authority, diagnostic imaging, and a “quick fix” to resolve their pain complaint. However, patients are subjected to long waiting times, and PHC staff describe that patients are therefore often left to navigate the healthcare system on their own. As a result, the responsibility for seeking care is mainly placed on the patients themselves, without any guidance regarding appropriate care paths.

#### In search of the silver bullet

This category reflects PHC staff’s perception of how patients experiencing back and neck pain often search for a *“Silver Bullet”* - a definitive diagnosis, a powerful prescription, or a “quick fix”. PHC staff perceive that patients prefer this to be delivered by the physician. In addition, patients tend to gravitate towards visiting physicians and their practices.

There seem to be a wish from patients to receive some form of imaging procedure for their complaint, most often MRI. They have probably heard from someone they know who got one and there is probably some kind of fear among patients that there is something wrong with them. They are worried that it could be something serious, hence they want to visualize it via diagnostic imaging. This results in me having to spend much time explaining to these patients that imaging is not always required.

(PHC_1_, physician)

*Patients often reject the idea of not receiving an examination by the physician and not having a radiological study performed.* (discussion in PHC_2_)

#### Left waiting alone

The category entitled *“Left Waiting Alone”* reflects health professionals’ perception of patients having to wait for a long time before they receive care and are, therefore, often left to self-manage. Although participants expressed that patients should be responsible, the current approach to back and neck pain risks having patients feel unsupported and without guidance.

*I think the management is working all right. As you say; many want to see a doctor first*,* which is where the process gets stuck. Even though it has gotten better over the years with patients rehabilitating themselves for these complaints. They are often sent for physiotherapy by the PHC*,* verbally or with a referral. And we do try to spread the recommendations between the physiotherapist and the chiropractor*,* as we have that available to us*,* too. To even out the waiting times.*

(PHC3, physiotherapist)

### Theme 2: Reconfiguring the care process

Staff at PHCs recognize both the limitations of the current system for dealing with patients presenting with back and neck pain and the potential of licensed chiropractors and naprapaths to improve the current management. The participants articulated a desire to reduce waiting times, lower the number of unnecessary diagnostic procedures, low-value care, and decrease the workload on physicians. Manual therapists were described as potential contributors to these goals. However, a lack of knowledge about their skills, uncertainty about their roles, and concerns about practical issues such as limited physical space and the sheer volume of patients were identified as barriers to implementation.

#### Bridging the gap

This category reflects the unfulfilled potential that licensed chiropractors and naprapaths may have within PHC. Participants expressed that they and patients alike long for clarity and trust in manual therapists’ work. Chiropractors and naprapaths are seen as valuable, yet somewhat unknown clinicians. Participants felt like increasing knowledge about and confidence in the role of licensed chiropractors and naprapaths could bridge this gap. Pursuing this further, during one group discussion, participants suggested collaborating via scheduled visits and organizing lectures from different professions to exchange knowledge and learn more about one another (PHC_1_, discussion)

*I think patients need to visit [manual therapists] more. Once they see them and realize that they received good care that helped them*,* they will also understand that they don’t always need to see the doctor. I think it might be a question of different generations. Once implemented*,* this strategy will demonstrate to patients that it works.* (PHC_1_, nurse)

I think that it is crucial to establish a good collaboration with a manual therapist in this role. This is really of great importance. That this person knows what we do as physicians and that we know when we should refer patients to the manual therapist

(PHC1, physicians)

#### Easing the burden

Participants perceived licensed chiropractors and naprapaths as a potential solution to an overloaded healthcare system. Their involvement could relieve strained physicians, reduce the number of unnecessary visits, and prevent low-value care. All in all, potentially creating a smoother and more efficient care process. When asked about potential incentives of having manual therapists in a triaging function of patients with back and neck pain in the PHC, two physicians answered that it would reduce the number of sick leave notes, medical pain prescriptions, and reduce the number of visits to the doctor (PHC_3_).

When asked if they thought licensed manual therapists could reduce their workload, one physician answered:

Without a doubt! We have a lot of things that we need help with and there is always a lack of available time slots for physicians. Additionally, there is not always much we as physicians can do for these patients. I think that in many cases, manual therapists are often better at diagnosing back and neck pain compared to physicians.

(PHC1, physician)

#### Avoiding low-value care

In this category, participants expressed an understanding of the difficulties related to the uncertainty of back and neck pain diagnosis, and how this may lead to the use of low-value care and overreliance on the physician’s competence.

*/…/ there is not always a clear explanation for back pain and in these cases I think it would be advantageous for patients to receive a thorough physical examination along with a clear description about their issue. Such as*,* what they can expect and how they should act in relation to their pain which in turn could reduce the risk of them seeking care multiple times.* (PHC_1_, resident)

*There may be a certain…dominance in that they [patients] want x-rays when there is no need*,* and one has to sit there and explain to them that this is unnecessary and that other interventions should be tried first.* (PHC_2_, physician)

#### Space and time as precious commodities

In this category, participants highlighted structural limitations that currently exist within the PHC, including a lack of physical space and strenuous workloads, which make implementing new roles difficult. As a result, the practicalities of accommodating licensed chiropractors and naprapaths become barriers. One participant simply said:

There are no rooms here. (PHC2, physician)

/…/ perhaps a manual therapist could have a triaging role over the phone or via a chat function because there is no way a single manual therapist in a triaging role could manage all patients with physical visits as they account for roughly 15% of all visits. (PHC3, physician)

### Theme 3: Challenging boundaries of professional identity

This theme describes the ongoing negotiation within the PHC community regarding professional roles. Staff expressed concerns about the overlap between the roles of licensed chiropractors and naprapaths and physicians, including fears that manual therapists might encroach upon responsibilities traditionally held by physicians. Participants’ perception of the large variability in licensed chiropractors’ and naprapaths’ competencies further contributed to their uncertainty and skepticism. These beliefs challenged the development of mutual trust and collaboration, perhaps contributing to a hesitancy to fully integrate manual therapists into triaging roles for patients with back and neck pain.

#### Shaky foundation for future collaboration

*“Shaky Foundation”* captures the confusion and uncertainty seen by PHC staff regarding the implementation of licensed chiropractors and naprapaths at the PHC. Participants expressed concerns related to collaboration marked by limited communication between healthcare professionals, unclear understanding of each other’s roles, and uncertainty regarding the consistency of manual therapists’ clinical competence. Primary healthcare staff describe a landscape where variability in skill levels and a general lack of insight into licensed chiropractors’ and naprapaths’ training, knowledge, and capabilities raise questions about professional trust and integration. These viewpoints highlight the unstable terrain on which triage models involving manual therapists are being considered.

*My thought is that we*,* as health care professionals*,* are not in full agreement on who does what. Maybe the first step would be to set the framework of who does what and to whom I can refer patients?* (PHC_1_, nurse)

*I have no idea what chiropractors and naprapaths do. If I recall correctly*,* during my time in medical school we were taught that physiotherapists are more evidence-based in how they work compared to chiropractors and naprapaths. I usually say that if patients have experienced benefit from seeing a chiropractor or naprapath then go ahead*,* whatever floats your boat. But it is not something that I’m willing to recommend and feel comfortable with from an evidence-based perspective.*

(PHC2, physician)

#### Blurred professional lines

This category highlights staff perceptions of a significant overlap in expertise among different licensed chiropractors and naprapaths, accompanied by a lack of clarity regarding their distinct scopes of practice. Concerns also emerge around role encroachment, where manual therapists may assume responsibilities traditionally reserved for physicians. Furthermore, the fear that such role shifts could compromise patient safety, expressed explicitly through concerns about missing severe illnesses. When asked about the risk of missing serious illness, one participant expressed it would be *“scary to hand over responsibility for discharging back and neck pain patients”* (PHC_1_, physician).

The same physician then elaborated further by saying:

*I think adding a manual therapist in a triaging role for back and neck pain patients could be a good idea. However*,* there need to be proper protocols in place to handle red flag symptoms so that conditions like kidney stones or more serious illnesses are not missed as symptoms of these conditions are probably not as frequent for manual therapists as they are for physicians.* (PHC_1_, physician)

Thus, based on the lack of knowledge, there seems to be a hesitancy towards integrating licensed chiropractors and naprapaths in PHCs. Personal experience is varied, and the need for formal structures of a triaging function is vital.

## Discussion

This study contributes to a deep and nuanced understanding of the perspective of various professionals at the PHC involved in delivering care to individuals with back and neck pain. It explores the perspectives of physicians, physiotherapists, nurses, and management. Specifically, if the current management of patients with back and neck pain at these PHCs needed improvement, and perceptions towards the potential role of licensed chiropractors and naprapaths in triaging patients with back and neck pain at PHCs. Staff at three PHCs in Stockholm described **t**he current management of patients with back and neck pain in terms of navigating patients’ expectations: an expectation and overreliance from patients concerning seeing a physician and receiving imaging, not other healthcare professionals, resulting in long waiting times for physicians. When asked about assigning licensed chiropractors and naprapaths to triage these patients at the PHC, participants expressed that this would mean reconfiguring the care process, and that poor knowledge and concerns about the competence of manual therapists would challenge the boundaries of professional identity.

### Navigating the weight of expectations

Participants expressed that they believed that patients with back and neck pain often expected to see their physician, not other healthcare professionals, resulting in long waiting times for all patients to see a physician. An explanation could be their trust in physicians’ competence, but also that physicians represent the top of the health care hierarchy, which may help patients in their search for quick fixes. This search for the silver bullet may lead to resistance from patients towards other options, including consulting manual therapists, who may be viewed as less authoritative figures in the health care space.

Our findings underscore the pivotal role of trust in shaping the dynamics within the healthcare community. Patients’ perceived trust in physicians influences their expectations and preferences, reinforcing the established hierarchy and shared practices. In the context of Lave and Wenger’s theory of CoP [16], this trust is essential for mutual engagement, joint enterprise, and the development of a shared repertoire. The issue of trust has previously been identified as a barrier in incorporating chiropractors into interprofessional practice [25]. By understanding and addressing the trust dynamics, healthcare providers can work towards a more inclusive community of practice that values and integrates diverse expertise. Additionally, the concept of social learning, central to CoP, is relevant to “Navigating the weight of expectations,” as patients’ trust and preferences are shaped through their interactions and experiences within the healthcare community.

### Reconfiguring the care process

Participants related the introduction of licensed chiropractors and naprapaths as potentially changing the care process, leading to less use of low value care: reducing imaging, the number of sick leave notes and medical pain prescriptions, and more efficient care. In the US, such a change was tested as a standardized pathway for triaging patients with low back pain among members of the multidisciplinary team, resulting in good clinical outcomes at a low cost with high levels of patient satisfaction [26]. Although it was recognized that patients should self-manage to a higher degree, participants also acknowledged the need for guidance in this process.

The introduction of licensed chiropractors and naprapaths can reconfigure the care process through enhanced collaboration. Examining our results through the lens of CoP [16], collaboration is crucial for fostering mutual engagement and developing a cohesive approach to patient care. By promoting collaboration between licensed chiropractors, naprapaths and physicians, the healthcare community can reduce low-value care and support patients in self-management, leading to more efficient and effective care. Furthermore, the concept of identity, central to CoP [16], is also pertinent, as manual therapists and physicians develop their professional identities through their collaborative practices and interactions within the community.

### Challenging boundaries of professional identity

Participants expressed poor knowledge about licensed chiropractors and naprapaths competence and confusion concerning differences between them. Licensed manual therapists in Sweden (chiropractors and naprapaths) have been part of Swedish Health and Welfare system for more than 30 years, with a four- to five-year full-time evidence-based education aiming to educate health care providers with expertise in the management of persons with musculoskeletal problems and injuries. This includes differential diagnosis, advice, reassurance, treatment, rehabilitation, health promotion and disease prevention. However, this uncertainty may reflect a threat to professional identity, described for physicians as that of medical expert and care coordinator [27]. Adding manual therapists, not only as part of the team, but also as first contact for these patients, may challenge the boundaries of professional identity. In a recent study of physicians’ attitudes towards collaboration with pharmacists, they made it clear that professional boundaries should not be crossed [28]. However, ten years after the introduction of chiropractors in the US as Primary Spine Care Providers, a positive shift has been noticed in other professionals’ attitudes toward this role [29].

Concerns were raised regarding potentially missing serious pathology, as the triaging role involves handing over the gatekeeping responsibilities for patients with back and neck pain. This lack of trust is found in previous research of interprofessional collaboration in different settings [27, 28, 30].

These findings illustrate the challenges and opportunities in negotiating professional identities within the healthcare community. Drawing on Lave and Wenger’s work and scrutinizing our results through the perspective of CoP [16], integration, trust, and collaboration are crucial for fostering mutual understanding and effective healthcare delivery. Additionally, the concept of identity is significant here, as licensed chiropractors and naprapaths negotiate their professional identities and establish their roles within the healthcare community through ongoing participation and interaction. By promoting these elements, the healthcare community can better understand and utilize the competencies of licensed manual therapists, ensuring that patients receive comprehensive and effective care. Legitimate Peripheral Participation (LPP), a central tenet of CoP [16], is particularly relevant here. LPP describes the process by which newcomers, such as licensed chiropractors and naprapaths, gradually become full participants in the community. Through LPP, manual therapists initially engage in peripheral activities, gaining experience and building trust. Over time, as they demonstrate their competence and contribute to the community, they transition from the periphery to more central roles, becoming recognized and trusted healthcare team members.

### Relation to existing literature

A Canadian study [31] also investigated clinicians’ perspectives on the current management of patients with back and neck pain at PHCs. Like our participants, the Canadians knew that imaging is considered low-value care and results in high health system costs but also voiced that patients want validation from imaging.

We found that PHC staff expressed poor knowledge about licensed chiropractors and naprapaths’ competence. This is similar to another study from Sweden [32], where general practitioners reported having inadequate knowledge and minimal experience with chiropractic. This, together with an uncertainty regarding treatment effectiveness, led to a hesitancy towards recommending chiropractic to their patients.

In interviews concerning the use of manual therapies in the US, physicians expressed concerns such as limited patient awareness and practice autonomy [33]. The comment regarding physical space is also found in a previous study on professional boundaries among manual therapists [27]. It is worth noting that the concerns raised in our study may be those found when aiming for interprofessional collaboration in general. In an Australian study of attempting to integrate different health services, structural barriers and territorialism were mentioned [30].

Participants in a Swedish study [32] expressed uncertainty about manual therapists missing serious illnesses. A Norwegian study reported that general practitioners wanted information on examination findings, diagnosis, treatment, and advice given, when communicating with chiropractors about patients [34]. This could possibly be one way of mitigating this fear as expressed by the physicians.

### Methodological considerations

A purposeful sampling strategy was employed, which took various professional experiences into consideration. The participants had a variety of professions, ages, and working experiences. Further, the three PHCs had different locations, organizations, and exposure to manual therapists to produce experiences from a rich and representative sample.

Throughout the analytical process, and primarily due to the senior investigator’s prior understanding of the empirical context, continuous scrutiny of codes, categories, themes, and the original data transcripts was conducted to ensure a robust alignment between the data and the findings. We thus gave careful consideration to Patton’s dual criteria of internal homogeneity and external heterogeneity [35].

Selection of participants was left to the discretion of the PHC managers, and we only asked for a diversity of professions managing back and neck pain patients, which was achieved. However, we do not know if the included participants were selected based on other factors, such as experience (the range of experiences of our participants suggests that this is not the case), or if they were simply available at the time of the interview. It is difficult to judge the impact of this selection.

Another possible limitation of the study is that no repeat interviews were conducted, which was due to logistical constraints at the participating healthcare centers. This may have restricted opportunities for follow-up clarification and cross-case comparison.

The nature of small-scale qualitative research inherently limits the interpretation of our findings. Qualitative studies, which focus on detailed and in-depth analyses within the constructivist paradigm, differ significantly from large-scale population-based studies that operate within the post-positivistic paradigm. Consequently, generalizing the findings from qualitative research is neither feasible nor desirable. However, by providing a detailed description of the contextual setting, the participants involved, and the analytical process, along with drawing connections between our findings and relevant theories and existing literature, we aim to enable readers to assess the applicability and relevance of our findings to their contexts.

While focus groups provide a valuable forum for in-depth analysis of topics, particular sensitivity surrounding the management of patients with back and neck pain at PHCs may have impeded this approach. Some aspects of the phenomenon may be sensitive, making participants reluctant to share personal experiences in a group setting. Although semi-structured interviews might have been a more suitable alternative method, logistical constraints prevented their implementation.

In this study, member checking, such as giving feedback on transcripts and findings, was not utilized, aligning with the cautionary stance of several methodologists [36, 37]. The decision was based on three primary considerations. Firstly, member checking assumes a fixed truth or reality, which may not align with the interpretive nature of qualitative research. This assumption can be problematic as qualitative research often seeks to explore multiple perspectives and understandings. Secondly, individual participants might struggle to grasp the broader context derived from data collected from multiple sources, potentially limiting their ability to provide meaningful feedback. Lastly, the process of member checking requires significant time and logistical resources, which can be challenging to manage effectively. These factors collectively informed our decision to employ alternative methods to ensure the credibility and reliability of our findings.

Existing literature suggests that patient satisfaction tends to be lower in gatekeeping systems compared to direct-access systems [38]. It is imperative to understand patients’ perceptions of licensed chiropractors and naprapaths in this gatekeeping role, as well as their willingness to accept manual therapists as primary contact providers. Addressing this knowledge gap is essential if licensed chiropractors and naprapaths are to be successfully implemented in PHCs. Future research should focus on incorporating qualitative data to explore patient experiences and satisfaction with manual therapists functioning as gatekeepers.

### Perspectives on licensed chiropractors and naprapaths as gatekeepers in phcs

We are currently planning a study in Stockholm of licensed chiropractors and naprapaths as “gatekeepers” at PHCs to explore patient outcomes, use of low-value care, and PHC workload, since there is a scarcity of empirical evidence or detailed studies on the effectiveness of manual therapists as gatekeepers in PHC. The literature suggests that gatekeeping can positively impact the quality of care, health outcomes, and healthcare utilization [39]. Insights into the perceptions of PHC staff towards manual therapists in a triaging role for patients with back and neck pain within the PHC team are crucial for conducting such a study. We learned that PHC staff must be thoroughly educated about licensed chiropractors’ and naprapaths’ competence prior to introduction in a PHC. This would also mitigate the concern that manual therapists would miss severe pathology. Among the incentives for licensed chiropractors and naprapaths to have a triaging role, decreased physician workload and more patient choice were mentioned. The concerns included practical issues (time and space).

## Conclusion

Staff at three primary healthcare centers in the Stockholm area described the current management of patients with back and neck pain as *navigating the expectations of patients*, in terms of their overreliance on seeing their physician, long waiting times, and expecting imaging. These issues were seen as incentives for licensed chiropractors and naprapaths to triage these patients and as an opportunity to improve care. A lack of knowledge about the competence of manual therapists was a concern, and assigning licensed chiropractors and naprapaths to the primary healthcare team would necessitate a reconfiguration of the care process, potentially challenging the *boundaries of professional identity*.

## Supplementary Information

Below is the link to the electronic supplementary material.

Supplementary Material 1

Supplementary Material 2

## Data Availability

The data generated and analyzed during the current study are not publicly available due to the risk of breaching study participants’ data anonymity.

## Declarations

### Author information

JH is a full-time medical student. ES is a full-time PhD student. Associate professor PJP is a full-time researcher/lecturer at Karolinska Institutet. Associate professor IA is a full-time researcher at Karolinska Institutet and ELIB. Professor EvS is a full-time researcher at Sophiahemmet Högskola and Karolinska Institutet. Assistant professor AE works part-time as a researcher at Karolinska Institutet (20%), at the Nordic Institute of Manual Medicine (NIMM) (20%), and as deputy clinic manager at two multi-disciplinary rehab units (60%).

## Abbreviations

CoP: Communities of Practice
LPP: Legitimate Peripheral Participation
MT: Manual therapist
PHC: Primary Healthcare Center
