# Supplementary material for: Gatekeepers in primary care - a qualitative study on manual therapists’ role in triaging patients with back and neck pain
Source: BMC Health Serv Res. 2025 Oct 27;25:1413. doi: 10.1186/s12913-025-13593-w (PMC12560454; doi:10.1186/s12913-025-13593-w)
Supplement: Supplementary file 1 — Supplementary Material 1 [file 12913_2025_13593_MOESM1_ESM.docx]

Appendix 1: Descriptive data of 20 participants in a focus group study on staff of three PHCs in the Stockholm region.

| **Participant** | **Age** | **Sex** | **YP** | **YW** | **Profession** |
| --- | --- | --- | --- | --- | --- |
| 1 | 39 | M | 10.5 | 4 | Physician |
| 2 | 30 | M | 2 | 1 | Nurse |
| 3 | 27 | M | 1 | 0.1 | Nurse |
| 4 | 27 | F | 2.5 | 0.4 | Intern (medicine) |
| 5 | 46 | F | 20 | 6 | Nurse |
| 6 | 59 | F | 20 | 5 | Nurse |
| 7 | 36 | F | 11 | 6 | Nurse |
| 8 | 52 | M | 20 | 7 | Physician |
| 9 | 52 | F | 12 | 1 | Physician |
| 10 | 60 | F | 4 | 3,5 | Associate nurse |
| 11 | 30 | F | 5.5 | 0,5 | Resident (medicine) |
| 12 | 39 | F | 14.5 | 2,5 | Nurse |
| 13 | 32 | F | 5 | 1 | Resident (medicine) |
| 14 | 41 | F | 15 | 2 | Physician |
| 15 | 30 | F | 12 | 12 | Physiotherapist/manager |
| 16 | 41 | M | 14 | 12 | Physician |
| 17 | 47 | M | 15 | 5 | Nurse |
| 18 | 56 | M | 15 | 8 | Nurse |
| 19 | 70 | F | 30 | . | Physician |
| 20 | 56 | M | 22 | 5 | Physician |

**Age**= Participant age; **M**= Male; **F** =Female; **YP** =Years in practice; **WP**= Years at workplace. **Intern**= a medical student in their final phase of education, **Resident**= a medical doctor in specialist training.
