# Supplementary material for: Gatekeepers in primary care - a qualitative study on manual therapists’ role in triaging patients with back and neck pain
Source: BMC Health Serv Res. 2025 Oct 27;25:1413. doi: 10.1186/s12913-025-13593-w (PMC12560454; doi:10.1186/s12913-025-13593-w)
Supplement: Supplementary file 2 — Supplementary Material 2 [file 12913_2025_13593_MOESM2_ESM.docx]

# Interview guide, translated from Swedish.

Manual therapists in primary healthcare as first point-of contact for patients with back and neck pain: how does staff at primary healthcare centres perceive this scenario?

## Introduction

- Introduce the interviewer and the project aim.

- Practical information: time allotted, turn off mobile phones, a private room. We would like you to share your perceptions and attitudes.

- The interview will be recorded; data will be de-identified.

- Summarize the GATE project (that manual therapists at the primary healthcare centre could screen/triage patients with back and neck pain). It is of UTMOST importance that manual therapists are introduced properly: that they are authorized healthcare personnel with a specific competence regarding musculoskeletal conditions. IMPORTANT that the suggested role at the primary healthcare centre is purely to triage, not to treat, patients. The triaging will result in a recommendation that some patients see their physician (imaging, blood tests, specialist referral), or to another caregiver in primary care (physiotherapist or manual therapist), but the majority will merely need information about their condition, reassurance, support, and advice on self-management.

- Questions from participants.

## Background

- What profession do you belong to?

- Sex, age, and time working at this healthcare centre.

## Needs, incentives, concerns

-How are patients with back and neck pain managed today (processes/pathways, referrals, rehabilitation)?

- Do you perceive any difficulties/challenges regarding today’s management (waiting times, patients’ demands regarding sick leave or imaging, waiting time for specialist referral)?

- What do you know regarding the competence of manual therapists in diagnosing disorders such as back and neck pain?

- Do you think that a manual therapist could alleviate your workload? If so, how (fewer patients, shorter waiting timers, contribute with diagnostic input, different knowledge, saving resources…)?

- How could a manual therapist improve patient outcomes (outcomes, waiting time…)?

- What concerns do you perceive with manual therapists triaging patients with back and neck pain at the primary healthcare centre (legal, practical)?

- What incentives do you perceive with manual therapists triaging patients with back and neck pain at the primary healthcare centre (room, routines, an existing functioning triaging function)?

Finally

- Do you want to add anything to this discussion?

- May we get in touch for clarifications?

- Thank you for your participation.
